# Textile Functionalization Using LTA and FAU Zeolitic Materials

**DOI:** 10.3390/polym15010099

**Published:** 2022-12-26

**Authors:** Fabian N. Murrieta-Rico, Rosario I. Yocupicio-Gaxiola, Joel Antúnez-García, Armando Reyes-Serrato, Perla Sánchez, Vitalii Petranovskii

**Affiliations:** 1 Ingeniería Mecatrónica, Universidad Politécnica de Baja California, Mexicali 21376, Mexico; 2 Centro de Nanociencias y Nanotecnología, Universidad Nacional Autonóma de México, Ensenada 22800, Mexico

**Keywords:** zeolites, LTA, FAU, textile modification, XRD, UV-Vis, oligodynamic

## Abstract

COVID-19 has drawn worldwide attention to the need for personal protective equipment. Face masks can be transformed from passive filters into active protection. For this purpose, it is sufficient to apply materials with oligodynamic effect to the fabric of the masks, which makes it possible to destroy infectious agents that have fallen on the mask with aerosol droplets from the air stream. Zeolites themselves are not oligodynamic materials, but can serve as carriers for nanoparticles of metals and/or compounds of silver, zinc, copper, and other materials with biocidal properties. Such a method, when the particles are immobilized on the surface of the substrate, will increase the lifetime of the active oligodynamic material. In this work, we present the functionalization of textile materials with zeolites to obtain active personal protective equipment with an extended service life. This is done with the aim to extend the synthesis of zeolitic materials to polymeric fabrics beyond cotton. The samples were characterized using XRD, SEM, and UV-Vis spectroscopy. Data of physicochemical studies of the obtained hybrid materials (fabrics with crystals grown on fibers) will be presented, with a focus on the effect of fabrics in the growth process of zeolites.

## 1. Introduction

As is well known, the current spread of diseases attracted worldwide attention; this is mainly attributed to the apparition of COVID-19, as well as the expected emergence of new pandemics in the near future. This leads to the need for personal protective equipment, such as face masks. These devices can be transformed from passive filters into active protection. For this purpose, it is enough to apply materials with an oligodynamic effect on the fabric of the masks, which makes it possible to destroy infectious agents that have fallen on the mask with aerosol droplets from the air stream [1].

Modification of polymers and textiles with nanoparticles (NPs) has been already reported. In particular, polymers have been modified with silica NPs for textile wastewater treatment [2]. In other case, carbon cloth was modified with Ag, as a result, the modified textile was found to work as a triboelectric nanogenerator [3]. Although these applications are interesting, in the recent years, biocidal properties of some nanomaterials have gained attention. In case of textiles for antimicrobial purposes, the use of NPs has been studied. In this regard, cotton fabrics modified with Ag NPs exhibited biocide effects against *Escherichia coli* and *Staphylococcus aureus* [4]. This kind of NPs has been stabilized on a polyester fabric, and antimicrobial activity was observed against *E. coli* and *S. aureus* [5]. ZnO NPs were used for modifying cotton fabrics, which exhibited antifungal activity *against Candida albicans*, *Aspergillus niger*, and *Aspergillus fumigatus* [6]. In the case of ZnO nanoparticles is important to remember that these NPs can be synthetized though green methods, which are environmentally friendly [7,8,9], and also, ZnO NPs have features such as biocompatibility and biodegradability [10]. With the aim for achieving antibacterial and hydrophobic properties, polydopamine (PDA) was deposited on cotton fabric. Then Ag NPs were used for modifying the surface of PDA. For this textile, antimicrobial activity was observed against *E. coli* and *S. aureus* [11]. Tetrapod like nanostructures made of ZnO have been reported to present biocide properties [12,13], and recently, cellulose fibers were functionalized with these kinds of NPs. Such textile materials exhibited enhanced antibacterial properties against *E. coli*, *S. aureus* and *Candida albicans* [14]. Novel methods for attaching the NPs to textiles are sought, in this sense, Ag Nps have been fixed on polypropylene textiles that have oligodynamic properties against *E. coli* [15]. The use of gamma radiation is another recent method for modification of cotton. In this case, cotton fabric was submerged into Ag/chitosan, Ag/polyvinylpyrrolidone, and ZnO/polyvinylpyrrolidone, then, the fabrics were irradiated with a gamma source. As a result, the modified textiles showed antimicrobial activity against *S. aureus* and *Pseudomonas aeruginosa* [16]. Polyurethane nanofibers modified with SiO_2_ were generated by electrospinning. This with the aim to obtain waterproof and breathable textiles [17]. In the work of Irfan et al. [18], electrospun membranes of polyvinylidene fluoride and polyvinyl pyrrolidone were modified with different amounts of Ag NPs. These membranes showed antimicrobial activity against *S. aureus*.

**Figure 1:**
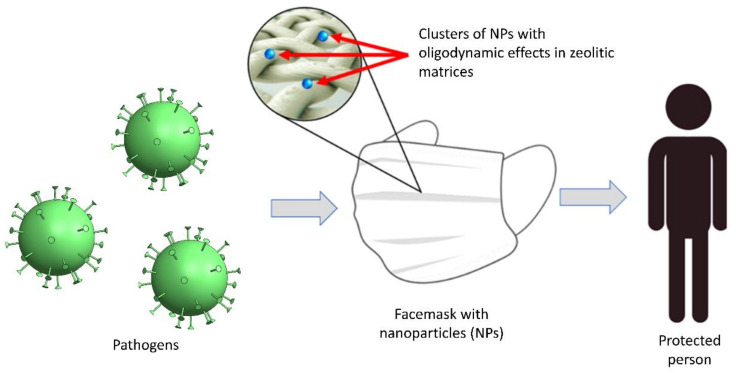
Basic elements in the application of zeolites for fabric functionalization.

Although zeolites themselves are not oligodynamic materials, they can serve as carriers for metal nanoparticles and/or compounds of silver, zinc, copper, and other materials with biocidal properties. When particles are immobilized on the surface of such a substrate, they are stabilized as compared to free particles and, consequently, the active oligodynamic material lifetime is increased; this basic idea is presented in Figure 1. For this reason, the use of a material such as zeolites to immobilize oligodynamic particles on a textile should be considered.

Although the use of zeolites for textile modification has already been reported, the relevant literature is not very voluminous, and is quite scarce in the case of fabrics modified with zeolites. For example, in [19], the modification of cotton and polyester fabrics after the application of clinoptilolite to them is presented. The aim of the experiment was to obtain a textile that protects against UV radiation and has an antibacterial effect. In the work of Carran et al. [20] zeolite A, both pure and exchanged with Na^+^ and Ca^2+^, was used for modification of wool-based fabric. Application of a zeolite showed no negative effects on stretching and other mechanical properties of this fabric. In addition, zeolite-based treatment has been shown to be a potentially efficient approach to improve surface hydrophilicity. Kunutoshi et al. [21] reported that zeolite A, and zeolite A loaded with Cu^2+^ were immobilized on a textile material, which was made of 100% cotton. Although the fabric modified with zeolite A did not show antiviral effects, in the case of fabric modified with Cu^2+^, it was capable to inactivate the Ck/Yamaguchi/7/04 H5N1 virus in a short incubation time, and also in the case when the virus was recovered immediately from the textile. In a similar way, Aguilar Palma et al. [22] reported the use of zeolite A for modification of a textile consisting of 100% cotton in this case, the Na+ ion was also replaced by Cu^2+^. The zeolite incorporated into the textile lasted for one dozen washings. In the work of Ojstršek et al. [23], different zeolitic phases (A, X, SOD, and MFI) were used for modifying a cotton fabric; as a result, thermal, optical and mechanical properties were reported. Another work reported on the use of copper ions (Cu^2+^) for the modification of LTA zeolite [24].

As a review of the available literature shows, the majority of fabrics used for modification with zeolites are made of wool and cotton. For this reason, the search for new applications of such functionalized fabrics requires the use of different zeolites on different fabrics. Accordingly, this work presents the functionalization of polymeric textile materials with zeolites that are useful for the development of active personal protective equipment with extended service life, with a particular focus on the effect of fabrics in the growth process of zeolites.

## 2. Materials and Methods

In this experiment, two different types of zeolites, FAU and LTA frameworks, were synthesized in the presence of different textiles in the reaction vessel. For each zeolite, the synthesis procedure followed the recipe provided by the IZA [24].

### 2.1. Synthesis of LTA Zeolite

In this case, deionized water, sodium hydroxide, sodium aluminate, and sodium metasilicate were used as starting materials; 80 mL of water and 0.723 g of sodium hydroxide were mixed until NaOH was completely dissolved. The mixture was divided into two equal volumes, which were stored in polypropylene bottles and labeled as “mixture A”. Half of mixture A was then mixed with 8.259 g of sodium aluminate; the mixture was stirred in the capped bottle until homogeneity was reached and labeled as “mixture B”. The other half of mixture A was mixed with 15.48 g of sodium metasilicate; the mixture was stirred until it reached homogeneity and labeled as “mixture C”. The two mixtures, B and C, were combined, and a thick gel was formed. This gel was stirred until a complete homogenization was observed. Prior to crystallization of the zeolite LTA, the gel was divided into four equal volumes, and a certain type of fabric was introduced into each one. Four types of woven fabrics were used: surgical pellon (100% polyester, fabric 1), poplin (65% cotton, 35% polyester, fabric 2), twine (100% polyester, fabric 3), and nonsurgical pellon (100% polypropylene, fabric 4), all of them from Mexico. Each fabric was stirred for 15 min in the gel. The crystallization process was then carried on; at this stage, each polypropylene vessel, containing the gel with the textile, was placed inside an oven for 4 h at 372 K. After the samples were withdrawn from the oven, they were cooled until they reached room temperature. Then, the contents of each vessel were mixed with 1 L of water, and this mixture was stirred for 30 min and then filtered; the fabric was also extracted. At this stage, it is important to note that the pellon-based fabrics were disintegrated, and only a powder was formed as a result of synthesis. Then, each fabric was stirred in 200 mL of deionized water for 30 min at 300 rpm. Each fabric, poplin and twine, was dried in an oven for 30 min at 372 K, and each powder was dried for 3 h at the same temperature. Finally, the prepared powders and fabrics were stored in separate containers.

### 2.2. Synthesis of FAU Zeolite

The synthesis procedure was started by mixing 100 g water and 100 g sodium hydroxide; the mixture was stirred until dissolved. The resulting solution was combined with 97.5 g of alumina trihydrate and stirred at 373 K until dissolved, and then cooled to 298 K. The resulting solution was mixed with 202.5 g of water. Next, 100 g of this solution was combined with 612 g of water and 59.12 g of sodium hydroxide. This solution was stirred until it was homogenized. In this way, precursor solution A was generated. Precursor solution B was prepared by mixing 219.7 g of sodium silicate solution, 612 g of water, and 59.12 g of sodium hydroxide until the mixture was completely dissolved. Solutions A and B were combined, and a gel was formed. The latter was stirred for 30 min at room temperature. The resulting gel was divided into four equal volumes, and a different fabric was immersed in each of them and stirred for 15 min. Then, the crystallization process was carried on; at this stage, each polypropylene vessel containing the gel with a fabric submerged on it was placed inside an oven for 8 h at 363 K. After the samples were withdrawn from the oven, they were cooled to room temperature. From here, the washing and drying process was the same as in the case of LTA zeolite; the pellon-based fabrics were also disintegrated, and the synthesis yielded only zeolite powder. As follows from this, when developing technologies for modifying tissues with zeolites, it is possible to discard the use of surgical pellon and twine, mainly because the fabrics are not capable of withstanding the pH in the liquor for zeolite synthesis.

### 2.3. Characterization

Both series of obtained powders and fabrics were characterized through by X-ray diffraction (XRD) by using Aeris Panalytical (Malvern, UK) equipment with Cu K alpha monochromatic radiation (λ=0.154056 nm, 40 kV, 15 mA) and UV-Vis spectroscopy by using a UV-Vis NIR Cary 5000 (Richmond CA, USA) spectrophotometer. Micrographs of the samples were obtained by using scanning electron microscopy (SEM) on JEOL JIB-4500 (Peabody, MA, USA) equipment.

## 3. Results and Discussion

The experiments yielded four powders and two fabrics for each zeolite: fabric 2 (poplin) and fabric 4 (non-surgical pellon). This is because the other two fabrics (surgical pellon and twine) were dissolved in an alkaline (pH 14) gel. The fabrics are labeled as F2 and F4 for fabric 2 and fabric 4, respectively—in the case of synthesis with LTA zeolite, as A-F2 and A-F4, and in the case of modification with FAU zeolite, as X-F2 and X-F4. All the samples generated from this experiment are listed in Table 1.

**Figure 2:**
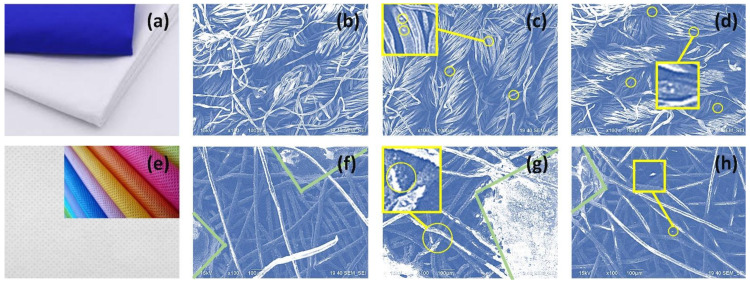
Fabrics used in the experiments: representative photograph of commercial F2 (**a**), micrograph of clean F2 (**b**), FAU-modified F2 (X-F2) (**c**), LTA-modified F2 (A-F2) (**d**), representative photograph of commercial F4 (**e**), micrograph of clean F4 (**f**), FAU-modified F4 (X-F4) (**g**), LTA-modified F4 (A-F4) (**h**).

From the SEM micrographs in Figure 2, the morphology of the particles in the powder can be seen, and a direct comparison of fabrics prior to and after the modification with zeolites is presented.

In Figure 2, the modification of fabrics after the chemical process can be observed. In the case of F2, the clean fabric (Figure 2b) shows a characteristic morphology that after chemical modification, with LTA zeolite (Figure 2c) and FAU zeolite (Figure 2d), is preserved. However, in both cases (X-F2 and A-F2), small particles are present on the surface of the threads, which can be confirmed in the insets of Figure 2c,d. This confirms chemical modification of the fabric by zeolite particles as a result of synthesis; a higher density of particles is observed in synthesis of FAU zeolite (Figure 2c). In the case of F4 (Figure 2f), the density of threads is lower than in F2, but there are square-shaped cavities that can be attributed to the original pattern of the fabric (areas delimited by green lines in Figure 2f–h). In this regard, such cavities seem to act like containers of zeolitic material for both zeolites, X-F4 and A-F4 in Figure 2g,h, respectively. In the case of zeolite X growth on F4 fabric, besides accumulation in the squared cavities, there is a change in brightness (Figure 2g) of most filaments compared to clean F4 (Figure 2f). This effect is related to the response of the surface to the incident electrons during the analysis. It is probably an effect of a process similar to mercerization when cotton is exposed to alkali [25]; however, in this case, the fabric consists of 100% polypropylene. Because the response of materials has obviously changed, we can assume that there is a growth of zeolite inside the threads of F4. In this sense, we can conclude that the area between the threads promoted crystallization.

In addition, particles are grown in threads of both fabrics, which can be confirmed in the insets of Figure 2g,h. Similar reasoning can be done for zeolite A in F4 (Figure 2h). Since both zeolites have different morphology, crystal growth follows a certain scenario, which can be assessed at the zones with the highest accumulation of zeolitic material. In this case, these are the squared zones in F4 (Figure 2g,h). After direct comparison, it can be noted that the zeolites in Figure 2g,h are different, which is expected for this experiment.

**Figure 3:**
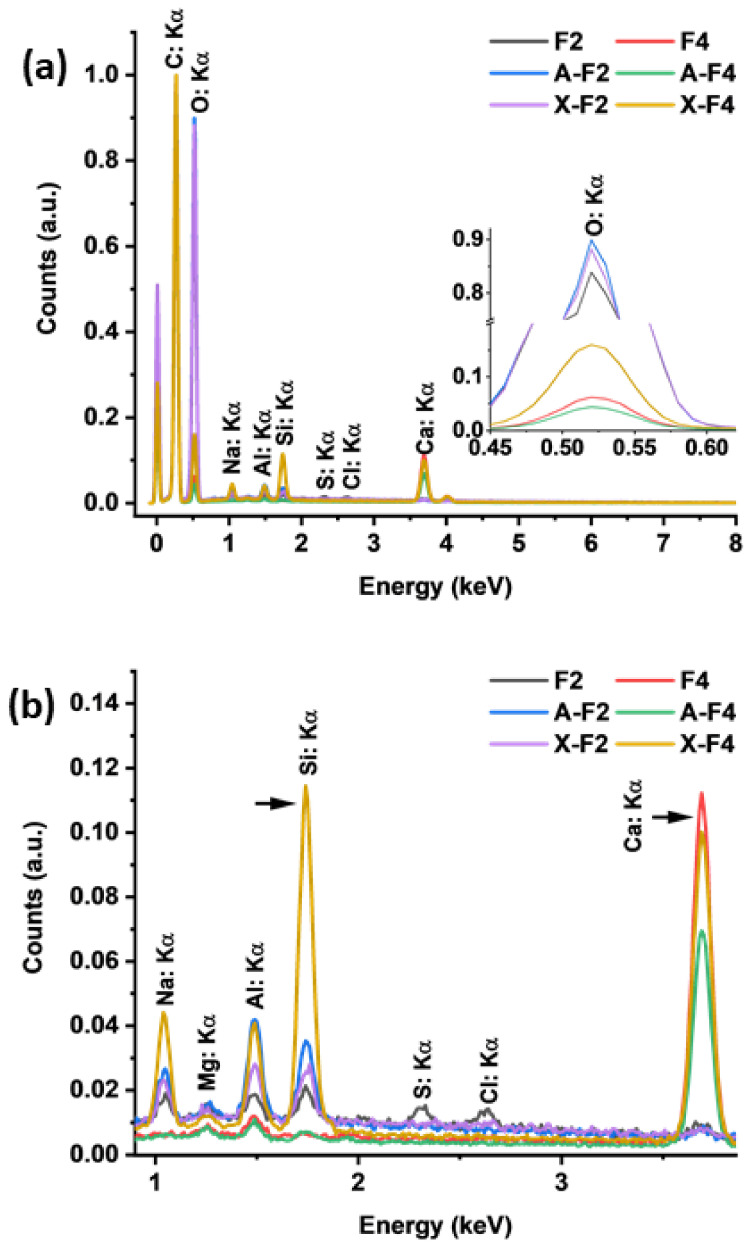
Analysis of elements in fabrics by the EDS: (**a**) full analysis from 0 to 8 keV, (**b**) zoomed region from 1 to 4 keV.

The chemical composition of the samples can be assessed by the EDS method. For fabric 2, poplin is known to contain cotton and polyester; the presence of C, O, and H, respectively, is expected [26,27]. Although EDS does not provide information on the latter, the presence of C and O is confirmed (see Figure 3a). As a result of the synergistic effect of cotton and polyester, a high C and O content is observed. In addition, Na, Mg, Si S, Al, Cl, and Ca are present in F2. In the case of modification with zeolite A, sample A-F2, there is an increment in Na, Al, and Si and a decrement in S and Cl. The increment in Na, Al, and Si can be attributed to the apparition of zeolitic species on the threads of F2. At the same time, the alkaline treatment during synthesis removes S and Cl in F2. Finally, a slight decrement of Ca is observed in A-F2 compared to F2. In the case of X-F2, there is an increment in O, Na, Al, and Si compared to F2, as well as a decrement in Mg, S, Cl, and Ca. Comparing the apparition of Na, Al, and Si, it can be noted that the greatest increment is observed in A-F2 than in X-F2. This can be attributed to the chemical affinity of the fabric for the reagents during synthesis, the nucleation sites on F2, and the crystal size of the particles in F2 after chemical modification. In the case of F4, a high amount of C is expected, but also a lower content of O, as shown in Figure 3. In comparison to F2, F4 shows only C, O, Al, Mg, and Ca content. After modification with LTA zeolite, there is a decrement in O and Ca content, but also an increment in Si content, which can be attributed to the presence of the particles shown in Figure 2h. In the case of zeolite X, X-F4 shows a higher amount of Na, Al, and Si than A-F4, but also lower content of Ca than F4, which is presented in Figure 3b. This is due to the composition of F4; such an increment of zeolite-specific elements suggests that zeolite crystals have grown inside F4 threads. It is important to highlight that the fabrics used in this experiment are not of reagent grade. Therefore, it is expected for the fabrics to have traces of other chemical processes that occurred prior to this experiment. This could explain the presence of Ca, Cl, and Mg in the EDS data.

**Figure 4:**
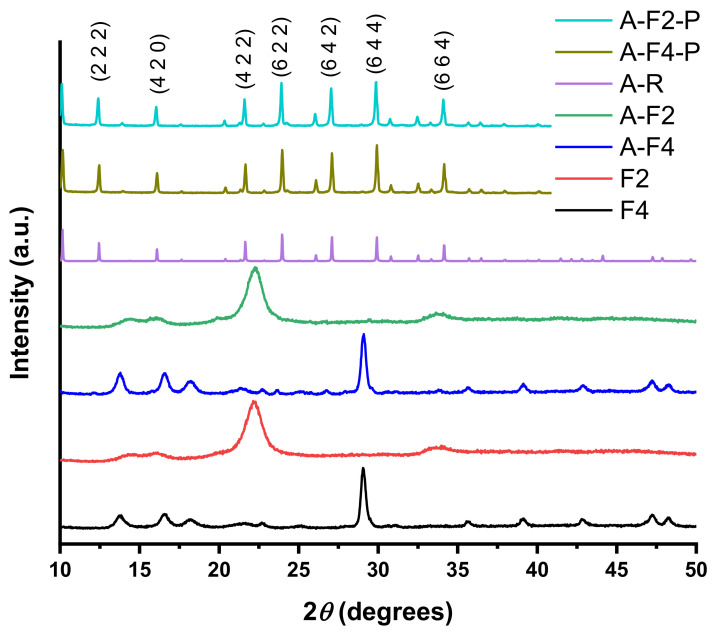
XRD-diffraction patterns corresponding to fabrics and zeolite A.

The XRD diffraction patterns of samples and powders obtained as a result of synthesis are presented in Figure 4. The diffractogram corresponding to fabric F2 shows the characteristic peak of cotton (0 0 2) at 2θ=22.74° [28], but the peaks at 14.96 and 16.5 degrees have a diminished intensity, which is likely an effect of inclusion of polyester in the fabric. The diffractogram corresponding to a polypropylene fabric (F4) shows the characteristic peaks reported by Niu et al. [29].

The powder and the modified fabric were recovered from the synthesis of both zeolites. In the case of LTA zeolite powders, for both fabrics, the diffractogram corresponds to the reference from IZA [24], which confirms the synthesis of the desired zeolite. In the case of F2, A-F2 and A-F4 show representative peaks of F2 and F4 in each case. However, although A-F2 is almost indistinguishable from F2, A-F4 shows the apparition of novel peaks when compared to F4. In this case, the are peaks at 21.6, 32.78, and 34.16 degrees. This can be attributed to the apparition of zeolitic crystals in F4.

**Figure 5:**
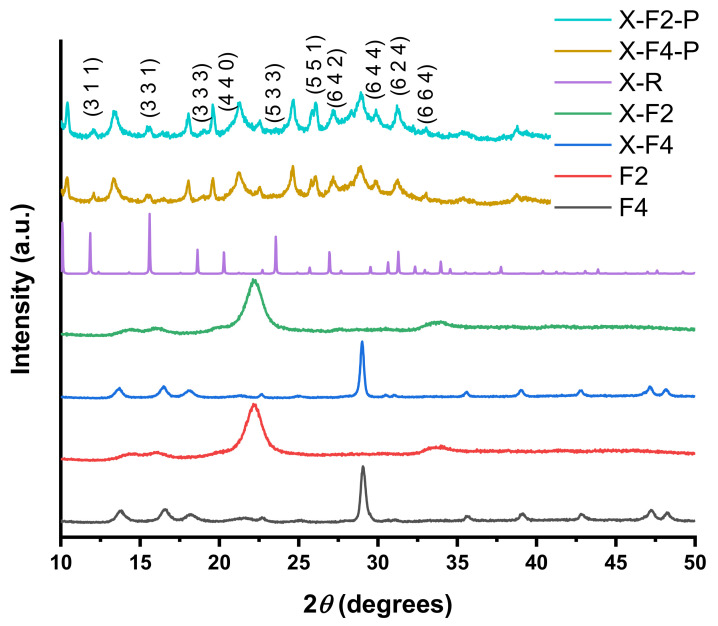
XRD-diffraction patterns corresponding to fabrics and zeolite X.

In the case of zeolite X, the diffractograms corresponding to powders and fabrics are presented in Figure 5. The XRD data of powders (X-F2-P and X-F4-P) show the presence of peaks corresponding to the reference diffractogram (X-R), but there are also other peaks that do not correspond to FAU zeolite, in which case we can consider that FAU zeolite was synthesized, but other crystalline species were also generated; in this case sodalite peaks are observed. In particular, X-F2 shows peaks of X-F2-P at 29.78, 33.67, and 40.8 degrees. In the case of F4, X-F4 shows peaks of X-F4-P at 21.6, 22.7, 25.04, and 29.4 degrees. These characteristics peaks indicate the presence of synthetized zeolite X crystals on the fabric F2.

Other peaks that do not belong to zeolite X, according to IZA [24], are generated by sodalitic structures. This is a known fact for faujasite —that is, if synthesis conditions are not thoroughly controlled, other phases such as P, A, or sodalite could be generated [30]. The formation of a mixture of several phases is obviously caused by the fact that the synthesis was carried out in the presence of tissue, but further discussion of this interesting question is beyond the scope of this paper

**Figure 6:**
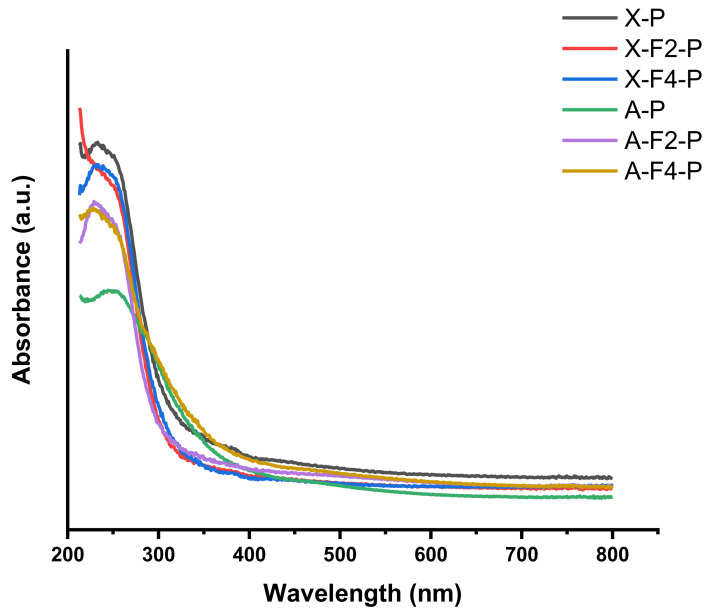
UV-Vis spectral data of samples generated during modification of fabrics.

In the case of the UV-Vis characterization, it is possible to estimate the response of the powders obtained from the synthesis with different fabrics. With this, we can get information about the properties of generated powders, and at the same time, study the effect of fabrics during the synthesis. As shown in Figure 6, the powders of all the samples have a maximum absorbance below 277 nm. In particular, the absorption of pure FAU powder has a local maximum at 232 nm. In the case of the powder generated during the modification of fabric F2, the local maximum of FAU is absent, which indicates that the generated powder has a lower adsorption. This is promoted due to the effect of fabric during the synthesis of zeolite X, which is a consequence of the variation in the crystalline structure obtained during synthesis. When F4 was modified with zeolite X, the peak at 277 nm of faujasite is present, but in general the absorbance of faujasite synthetized with F4 is lower than that of pure faujasite (X-P). It is also observed that at 346 nm, the absorbance of X-F2-P and X-F4-P converges, and almost identical values are observed up to 400 nm. According to Figure 6, the LTA powder has a local maximum at 249 nm. For these zeolites, with both fabrics, the absorbance increased, and the local maximum shifted to 230 nm. In particular, F2 generated a greater absorption in the material than F2, this is shown in Figure 6, after comparing A-F2-P and A-F4-P. At 290 nm, the LTA zeolites grown with F2 and F4 have the same absorption, and after this value both samples have a reliable difference in the exhibited absorbance up to 800 nm. Although the FAU (X-P) and LTA (A-P) zeolites have a quite different absorbance values that overlap (in the observed wavelength range, only at 292 nm), both fabrics generated zeolites that, above 665 nm, have almost the same absorbance value. Below of the crossing wavelength value, samples have a clear convergence among each type of zeolite, which is due to the properties of each type of zeolite. This can be attributed to the chemical modification generated by each fabric.

**Figure 7:**
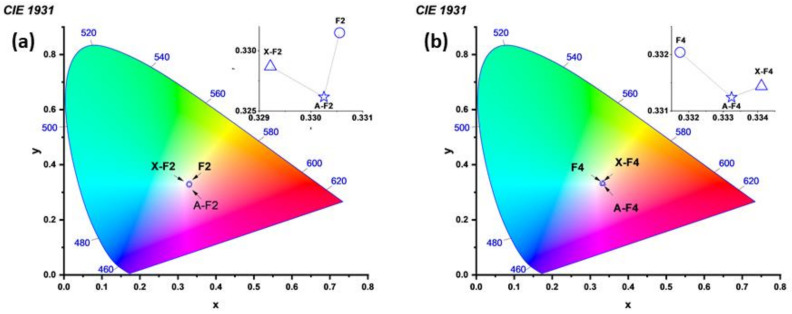
CIE Lab space evaluation of UV-Vis data from samples generated during modification of fabrics: analysis of fabric 2 (**a**) and fabric 4 (**b**).

The CIE Lab space was used for evaluating the color variations of F2 and F4 after the modification with FAU and LTA zeolite. The location of the point corresponding to the color exhibited for each sample is presented for fabric 2 and fabric 4 in Figure 7a,b, respectively. These points were generated after the use of UV-Vis data for each fabric. In the case of F2 (Figure 7a), the colors of F2, X-F2, and A-F2 are apparently overlapped, but if the zone corresponding to such points is zoomed, then the color variations can be evaluated. This is shown in the inset of Figure 7a. Both fabrics are apparently white at eyesight, but in the case of F2, the effect of LTA zeolite (A-F2) generates a variation of color toward a pink tonality; furthermore, in the case of F2 modified with FAU zeolite (X-F2), there is a variation of color in direction of green/blue tonalities.

In the case of fabric four (F4), the color corresponding to each sample seems to be almost the same (Figure 7b), but like fabric F2, after the observation of inset in Figure 7b, the variations of color can be evaluated. In the case of zeolite A, there is a variation of color in direction to pink tonalities. Moreover, after modifying of F4 with zeolite X, there is a variation of color in direction of red/orange tonalities.

Overall, F2 and F4 have quite similar colors, but although zeolite A caused almost the same effect in both fabrics, FAU zeolite generated the opposite effect in F4 when compared with F2.

## 4. Conclusions

In this work, a direct synthesis of LTA and FAU zeolites was carried out on two different fabrics. It has been shown that LTA grows even in the presence of fabrics, and FAU synthesis is complicated by the presence of fabrics. It has been observed that these fabrics are resistant to the alkaline conditions created during the crystallization process. As a result, zeolite growth showed no negative effects on stretching and other mechanical properties of fabrics. Direct growth was observed after the presence of zeolitic crystals on the fabric weave. In this sense, the procedure presented in this report allows us to obtain fabrics modified with LTA zeolites that are ready for modification with oligodynamic agents. Moreover, in case of zeolite X, the fabrics generate a change in the crystalline phases that are obtained, which can be used as a method for achieving novel synthesis conditions in the generation of alternative synthesis routes of zeolites. It is noteworthy that the fabrics generated in this study are intended for personal protective equipment, including application in disposable facemasks. This is done with the aim to cut off transmission chains of pathogens. As a future work, we expect to load zeolites with ions that offer oligodynamic capacity, and then evaluate the performance of polymeric fibers functionalized with zeolites after ion exchange.

## Figures and Tables

**Table 1:** Information of samples.

Label	Description
F2	Poplin: 65% cotton, 35% polyester
F4	Nonsurgical pellon: 100% polypropylene
X-F2-P	Powder of FAU zeolite generated during modification of poplin.
X-F4-P	Powder of FAU zeolite generated during modification of nonsurgical pellon.
A-F2-P	Powder of LTA zeolite generated during modification of poplin.
A-F4-P	Powder of LTA zeolite generated during modification of nonsurgical pellon.
X-F2	Poplin modified with FAU zeolite
X-F4	Nonsurgical pellon modified with FAU zeolite
A-F2	Poplin modified with LTA zeolite
A-F4	Nonsurgical pellon modified with LTA zeolite

## Data Availability

Not applicable.
